# The technology specialist: a 21st century support role in clinical care

**DOI:** 10.1038/s41746-019-0137-6

**Published:** 2019-06-26

**Authors:** Valerie A. Noel, Elizabeth Carpenter-Song, Stephanie C. Acquilano, John Torous, Robert E. Drake

**Affiliations:** 1 Westat, Inc., Lebanon, NH USA; 2 Department of Anthropology, Dartmouth College, Hanover, NH USA; 3 The Dartmouth Institute for Health Policy and Clinical Practice, Geisel School of Medicine at Dartmouth, Lebanon, NH USA; 4 Department of Psychiatry, Beth Israel Deaconess Medical Center, Harvard Medical School, Boston, MA USA

**Keywords:** Psychiatric disorders, Health services, Psychosis, Schizophrenia, Bipolar disorder

## Abstract

Mental health clinicians, clients, and researchers have shown keen interest in using technology to support mental health recovery. However, technology has not been routinely integrated into clinical care. Clients use a wide range of digital tools and apps to help manage their mental health, but clinicians rarely discuss this form of self-management in clinical interactions. This absence of communication is concerning because the safety and quality of the digital tools and apps people use may negatively affect their mental health outcomes. Mental health systems could benefit from someone to help identify technology-based supports that reflect current evidence and minimize privacy and security concerns. This technology specialist may also enhance the therapeutic bond between the client and the clinician. In working with a technology specialist, clients may begin to gain a sense of control over their mental health, and perhaps use fewer mental health services.

Computer technology has transformed how we manage our finances, relationships, and physical health. But what about our mental health? Mental health clinicians and researchers have shown keen interest in using technology to improve access, efficiency, and effectiveness of services.^1,2^ Some clinicians offer video-based office visits. Others collect data from clients through electronic prescreen assessments. Researchers are developing apps for symptom monitoring, mindfulness-based therapies, and other behavioral interventions targeting activities of daily living, substance use, and physical health.^3–11^ The promises crafted by technology innovators to improve mental health outcomes are widespread, but these digital tools may not be panaceas. Instead, they should be used to complement care. The critical question is how should we sustainably and effectively incorporate these tools into the mental health care system?

The disability resulting from serious mental illnesses (SMI), such as, psychosis, bipolar disorder, and major depression, is apparent at both the individual level, on physical, cognitive, social, and financial functioning, and societal level economics.^12^ Integrated approaches to treatment are the most effective forms of treatment and researchers have looked to technology to enhance and deliver these interventions. Research-developed apps for mental and physical health management for people with SMI have empirical evidence for their acceptability and effectiveness. Use of illness management apps for people with SMI, such as FOCUS and weCope, has shown reductions in psychotic and depressive symptoms, and improvements in general functioning;^13,14^ people with SMI have shown high rates of engagement with WellWave, an app which promotes physical activity;^6^ people with a first episode psychosis who used PRIME, a motivational enhancement app, for 3 months reported fewer depressive symptoms and increased self-efficacy.^15^

Nonresearch-derived mental health apps make up the majority of publically available mental health apps. Interventions such as PeerFit highlight how publically available devices and apps may enhance clinical interventions. PeerFit, a weight loss intervention for people with SMI, incorporates wearable devices (e.g., Zip^TM^ (FitBit [San Francisco, CA]) or Fuelband (Nike Inc. [Beaverton, OR]) and social media to increase participant engagement in peer-to-peer support.^16^ In another example, a client used a digital tally counter to track the frequency of auditory hallucinations during a medication dosing change^17^ (a simple alternative to the research-derived app, ClinTouch, which prompts users randomly throughout the day to report mood and psychotic symptoms.^9^) The data collected by the client not only informed dosing decisions but motivated the client to adhere to the medication. Despite these promising outcomes, wide-scale implementation of devices and apps in mental health care settings has not yet been realized.

Mental health centers may need a new support role in the delivery of mental health services that incorporate technology.^18–20^ We term this new role the technology specialist. People are already using mobile apps independently to support their mental health recovery (i.e., gaining a sense of control over illness-management, meeting personal needs, striving toward ones goals) but most do not discuss the use of apps in clinical sessions,^18,21^ even though the safety and quality of the apps they choose may affect their mental health outcomes.^22^ The technology specialist would use an individualized approach to identify and review electronic resources (e.g., websites, electronic devices, and apps) that may support a client’s specific recovery goals (e.g., a sleep cycle-monitoring app or a guided meditation app for someone who wants to improve their sleep). A broad yet structured search would include understanding features and identifying the required operating system, storage, and internet access.^23^ Further, it would evaluate the terms and conditions of privacy, sharing of personal information, and security.

We envision the work of the technology specialist as akin to a reference librarian, who assists in the identification of relevant digital tools and apps, thus laying the groundwork for productive discussions of mental health and technology between clients and members of the clinical team. We propose that making meaningful use of the technology in support of recovery should remain the shared responsibility of the client and clinician. For example, a clinician’s primary question to a client who is using a wearable fitness device would be, how active have you been? Or how many steps do you average per day? Not, how often are you wearing it? The fundamental focus is progress in recovery, not the tool itself. The clinician should help ensure the tool is supporting the recovery goal and the technology specialist can reinforce this purpose through brief contacts with the client and the clinician. We advocate that this work be guided by a shared-decision making approach to developing recovery goals, selecting digital tools and apps, as well as setting expectations regarding duration and patterns of use (e.g., daily, weekly, as needed, etc.) to maintain client engagement. For clients who struggle to meet engagement expectations, the technology specialist may place more emphasis on digital tools or apps that provide immediate feedback in response to usage.

Who would excel as a technology specialist? Having the patience to provide technical support, an interest in mental health, fluency in learning new technology, and the capacity to be creative are assets. A technology specialist could be a separate position or the activities could be subsumed in other clinical roles, such as peer workers or case managers. Strategies for successful implementation of a technology specialist in a mental health setting include securing diverse private and public funding streams from local and federal sources, committed agency leadership, formalized integration of the technology specialist services by establishing standardized workflow procedures, and ongoing consultation/supervision from digital mental health researchers and clinicians.^24^

We foresee many potential benefits of this new role. The technology specialist may enhance the therapeutic bond between the client and the clinician by framing the interactions on identifying an area of recovery, planning, and monitoring progress, thereby creating a structured course for therapy with a clearly defined end goal. Clients may begin to gain a sense of control over their mental health, perhaps using fewer mental health services. Clinicians may practice more effectively by using the data provided through these tools. The coupling of these foreseeable outcomes enhances a recovery-oriented mental health care system. The technology specialist in mental health care may facilitate systemic changes that align with the ever-changing landscape of health technology.
